# Impact of CYP2C19 metabolizer status on esophageal mucosal inflammation, acid exposure, and motility among patients on chronic proton-pump inhibitor therapy with refractory symptoms of gastroesophageal reflux disease

**DOI:** 10.1093/jcag/gwae005

**Published:** 2024-02-10

**Authors:** Cheng-Chun Tai, Samantha Medwid, Keith Mclntosh, Nilesh Chande, Richard B Kim, James Gregor

**Affiliations:** Department of Medicine, Schulich School of Medicine & Dentistry, Western University, London, ON N6A 5C1, Canada; Department of Medicine, Schulich School of Medicine & Dentistry, Western University, London, ON N6A 5C1, Canada; Division of Clinical Pharmacology, Department of Medicine, Schulich School of Medicine and Dentistry, Western University, London, ON N6A 5C1, Canada; Department of Medicine, Schulich School of Medicine & Dentistry, Western University, London, ON N6A 5C1, Canada; Division of Gastroenterology, Department of Medicine, Schulich School of Medicine & Dentistry, Western University, London, ON N6A 5C1, Canada; Department of Medicine, Schulich School of Medicine & Dentistry, Western University, London, ON N6A 5C1, Canada; Division of Gastroenterology, Department of Medicine, Schulich School of Medicine & Dentistry, Western University, London, ON N6A 5C1, Canada; Department of Medicine, Schulich School of Medicine & Dentistry, Western University, London, ON N6A 5C1, Canada; Division of Clinical Pharmacology, Department of Medicine, Schulich School of Medicine and Dentistry, Western University, London, ON N6A 5C1, Canada; Department of Medicine, Schulich School of Medicine & Dentistry, Western University, London, ON N6A 5C1, Canada; Division of Gastroenterology, Department of Medicine, Schulich School of Medicine & Dentistry, Western University, London, ON N6A 5C1, Canada

**Keywords:** CYP2C19, metabolizer status, proton-pump inhibitor, gastroesophageal reflux disease, ambulatory pH study, high resolution esophageal manometry

## Abstract

**Background:**

The extent of disease severity remains unclear among CYP2C19 rapid and ultra-rapid metabolizers with refractory symptoms of gastroesophageal reflux disease (GERD) on chronic proton-pump inhibitors (PPIs).

**Aims:**

To determine the impact of CYP2C19 metabolizer status in relation to chronic PPI therapy with a focus on the extent of esophageal inflammation, acid exposure, and motor function.

**Methods:**

This retrospective study included 54 patients with refractory GERD symptoms who underwent *CYP2C19* genotyping for PPI metabolism, esophagogastroduodenoscopy, ambulatory pH study, and high-resolution esophageal manometry. Patients were divided into three groups: normal metabolizer (NM) group, intermediate metabolizer/poor metabolizer (IM/PM) group, and rapid metabolizer/ultra-rapid metabolizer (RM/UM) group. The Chi-square test was used to analyze categorical variables, and one-way ANOVA for comparing means.

**Results:**

Rapid metabolizer/ultra-rapid metabolizer (RM/UM) group more frequently had either Los Angeles grade C or D GERD (7/19, 36.8% vs 1/21, 4.8%, *P* = 0.011) and metaplasia of the esophagus (9/19, 47.4% vs 2/21, 9.5%, *P* = 0.007) when compared to the NM group. RM/UM group were more frequently offered dilatation for nonobstructive dysphagia (8/19, 42.1% vs 3/21, 14.3%, *P* = 0.049) and more exhibited a hypotensive lower esophageal sphincter (LES) resting pressure compared to the NM group (10/19, 52.6% vs 4/21, 19%, *P* = 0.026). All three groups exhibited comparable DeMeester scores when PPIs were discontinued 72 hours before the ambulatory pH study.

**Conclusion:**

CYP2C19 RMs and UMs on chronic PPI with refractory GERD symptoms exhibited greater esophageal mucosal inflammation, as observed both endoscopically and histologically, and more were found to have hypotensive LES resting pressures and more were offered esophageal dilatation.

## Introduction

Gastroesophageal reflux disease (GERD) is one of the most common gastrointestinal diagnoses for outpatient visits,^1^ and its management using proton-pump inhibitors (PPIs) has been extensively established.^2,3^ Nevertheless, a subset of individuals experiences severe refractory diseases due to genetic factors linked to PPI metabolism.^3^ The metabolism of PPIs primarily occurs through the action of CYP2C19.^3^ Still, some variance exists among different PPIs: first-generation PPIs, including omeprazole and pantoprazole are mainly metabolized by CYP2C19 with secondary involvement of CYP3A4, while lansoprazole is primarily metabolized by both.^4–6^ Second-generation PPIs, including rabeprazole and esomeprazole, are less dependent on CYP2C19 pathway.^5,6^ Dexlansoprazole, another second-generation PPI, shares a similar metabolic pathway to lansoprazole.^6^ Depending on the genotypic combinations, individuals can be categorized into five distinct phenotypes, including CYP2C19 normal metabolizers (NMs), intermediate metabolizers (IMs), poor metabolizers (PMs), rapid metabolizers (RMs), and ultra-rapid metabolizers (UMs).^6^

Previous investigations have consistently demonstrated an elevated risk of inadequate response to PPI therapy in individuals classified as CYP2C19 RMs and UMs with GERD.^6,7^ This suboptimal response is attributed to lower PPI serum concentrations from increased metabolism, thereby causing insufficient inhibition of acid secretion by gastric parietal cells.^6–8^ The Clinical Pharmacogenetics Implementation Consortium (CPIC) guideline for *CYP2C19* and PPI dosing provides recommendations for first-generation PPI doses based on different patient *CYP2C19* genotypes.^6^ However, it is important also to note that simply increasing the dosage of PPI may not necessarily result in significant improvement in acid reflux symptoms.^9^ Optimally treating individuals who are RMs and UMs remains a challenging task.^8^ Moreover, comprehensive investigations on the extent of disease severity are lacking in this population undergoing chronic PPI therapy, highlighting the need for further research in this area.

In this study, we sought to determine the impact of CYP2C19 metabolizer status in relation to chronic PPI therapy specifically focusing on the degree of esophageal inflammation, esophageal acid exposure, and esophageal dysfunction involving results obtained from esophagogastroduodenoscopy (EGD), ambulatory pH study, and high-resolution esophageal manometry (HRM).

## Materials and methods

### Study design

This was a retrospective study that collected data from health records at London Health Science Center in Ontario, Canada, from March 2018 to May 2023. During this period, a total of 201 patients, who had been on chronic PPI therapy for over 3 months with persistent symptoms (experiencing at least one of the following: heartburn, regurgitation, atypical chest pain, or dysphagia), were referred for *CYP2C19* genotyping. The subset of 54 patients who underwent all investigations, including *CYP2C19* genotyping, ambulatory pH studies (from which DeMeester scores were derived), and HRMs, were included in this study. The DeMeester score is a composite score recording the degree of acid reflux experienced by an individual throughout a 24-hour duration.^10,11^ HRM is a complementary diagnostic tool that thoroughly characterizes esophageal and lower esophageal sphincter function through dynamic pressure measurement.^12^

All patients underwent EGD as part of the GERD diagnosis process and data were acquired from EGDs conducted prior to *CYP2C19* genotyping, pH studies, and HRMs. In cases where multiple EGDs were performed, data were extracted from the most recent EGD prior to these studies. PPIs were discontinued 72 hours before the ambulatory pH study; this is a part of our center’s protocol that was initially designed to objectively quantify GERD by eliminating any acid suppressants prior to the study. Data on PPI dosage and self-reported symptoms were collected on the day of *CYP2C19* genotyping to mitigate potential observer bias since PPIs were neither prescribed nor adjusted for patients on that day. The 54 patients were divided into three groups: the NM group (control group), the IM/PM group, and the RM/UM group. This study was approved by the Institutional Review Board at Western University. All patients provided written informed consent.

### *CYP2C19* genotyping

Whole blood samples were collected, and DNA was extracted using MagNA pure compact instrument (Roche). Genotyping was assessed using TaqMan allelic discrimination Assay (Thermo Scientific) for CYP2C19*2 rs4244285 (assay ID; C_25986767_70), CYP2C19*3 rs4986893 (assay ID; C_27861809_1) and CYP2C19*17 rs12248560 (assay ID; C_469857_10) on a ViiA7 real-time polymerase chain reaction system (Thermo Scientific).

### Evaluation and analysis

All total daily PPI dosages were converted to omeprazole equivalents (OEs) for uniformity based on relative potencies to omeprazole, which were 0.23, 0.9, 1.0, 1.6, 1.8, 1.83 for pantoprazole, lansoprazole, omeprazole, esomeprazole, rabeprazole, and dexlansoprazole, respectively.^13,14^

From EGD, biopsies were taken from any area of the distal esophagus with endoscopic evidence of GERD per the Los Angeles (LA) classification,^15^ or any areas suspicious for metaplasia, or, in the absence of visual abnormalities, normal mucosa to evaluate for histologic evidence of intestinal metaplasia. Findings from EGD, including the endoscopic appearance of LA grade C or D GERD and histologic diagnosis from biopsy, were included. From the ambulatory pH study, six parameters (total number of reflux episodes, percentage of total time esophageal pH < 4, percentage of upright time esophageal pH < 4, percentage of supine time esophageal pH < 4, number of reflux episodes ≥5 minutes, and longest reflux episode in minutes) were used to calculate the final DeMeester scores.^11^ The following parameters were collected from HRM: basal lower esophageal sphincter (LES) resting pressure, distal wave amplitude (DWA), onset velocity, distal contraction integral (DCI), and distal latency (DL).

Data were presented as counts or mean ± standard deviation (SD). Categorical variables underwent chi-square testing, while means were compared using one-way ANOVA. The Tukey–Kramer post-hoc test was used to make comparisons between group means. Differences between the results were considered statistically significant if the *P*-value was <0.05. Ambulatory pH studies were performed using Digitrapper Versaflex pH monitors & catheters (Medtronic, Minneapolis, MN) and AccuView pH software V5.2. HRMs were performed using Manoscan High Resolution Manometry System (Medtronic, Minneapolis, MN) and Manoscan software V3.0. All statistical analyses were performed using IBM SPSS Statistics version 28.0 (IBM Co., Armonk, NY, USA) and Excel (Microsoft Co., Redmond, USA). Figures were illustrated using GraphPad Prism version 10 for macOS (GraphPad Software, www.graphpad.com).

## Results

### Patient characteristics

Baseline characteristics of the patients in the three groups were compared in Table 1. A total of 54 patients were enrolled in the study and divided into three groups. The NM group comprised 21 patients. The IM/PM group comprised 14 patients (13 IMs and one PM). The RM/UM group comprised 19 patients (18 RMs and one UM). No significant differences were observed in age, body mass index (BMI), sex, history of tobacco use, history of *Helicobacter pylori* (*H. pylori*) infection, and self-reported symptoms on the day of CYP2C19 *genotyping*, between the groups. Six different PPIs were used among our patient population: dexlansoprazole, esomeprazole, lansoprazole, omeprazole, pantoprazole, and rabeprazole. Only one patient in the RM/UM group received omeprazole, and no patients in the IM/PM group received rabeprazole. No differences were observed in the usage of different PPIs among the three groups.

**Table 1.** Patient characteristics.

Patient characteristics		NM group (*n* = 21)	IM/PM group (*n* = 14)	RM/UM group (*n* = 19)	*P*-value
Age (mean ± SD)		59.31 ± 13.53	50.87 ± 14.56	54.58 ± 14.41	0.220
BMI (mean ± SD)		30.15 ± 7.59	27.03 ± 5.95	27.82 ± 6.54	0.364
Female		14 (66.7%)	12 (85.7%)	14 (73.7%)	0.452
History of tobacco use		8 (38.1%)	3 (21.4%)	7 (36.8%)	0.545
History of *H. pylori* infection		3 (14.3%)	0 (0%)	0 (0%)	0.082
Self-reported symptoms (%)	Heartburn	16 (76.2%)	8 (57.1%)	15 (78.9%)	0.336
	Regurgitation	16 (76.2%)	10 (71.4%)	13 (68.4%)	0.858
	Atypical chest pain	10 (47.6%)	5 (35.7%)	9 (47.4%)	0.747
	Dysphagia	6 (28.6%)	7 (50%)	9 (47.4%)	0.345
PPI usage	Dexlansoprazole	5 (23.8%)	5 (35.7%)	8 (42.1%)	0.503
	Esomeprazole	2 (9.5%)	2 (14.3%)	1 (5.3%)	
	Lansoprazole	2 (9.5%)	2 (14.3%)	4 (21.1%)	
	Omeprazole	0 (0%)	0 (0%)	1 (5.3%)	
	Pantoprazole	8 (38.1%)	5 (35.7%)	4 (21.1%)	
	Rabeprazole	4 (19%)	0 (0%)	1 (5.3%)	

The baseline characteristics of the patients in the three groups were compared. Data regarding the usage of PPI and self-reported symptoms were collected on the day of *CYP2C19* genotyping. No significant differences were observed between the groups. BMI, body mass index; SD, standard deviation.

### OE total daily dose

**Figure 1.**
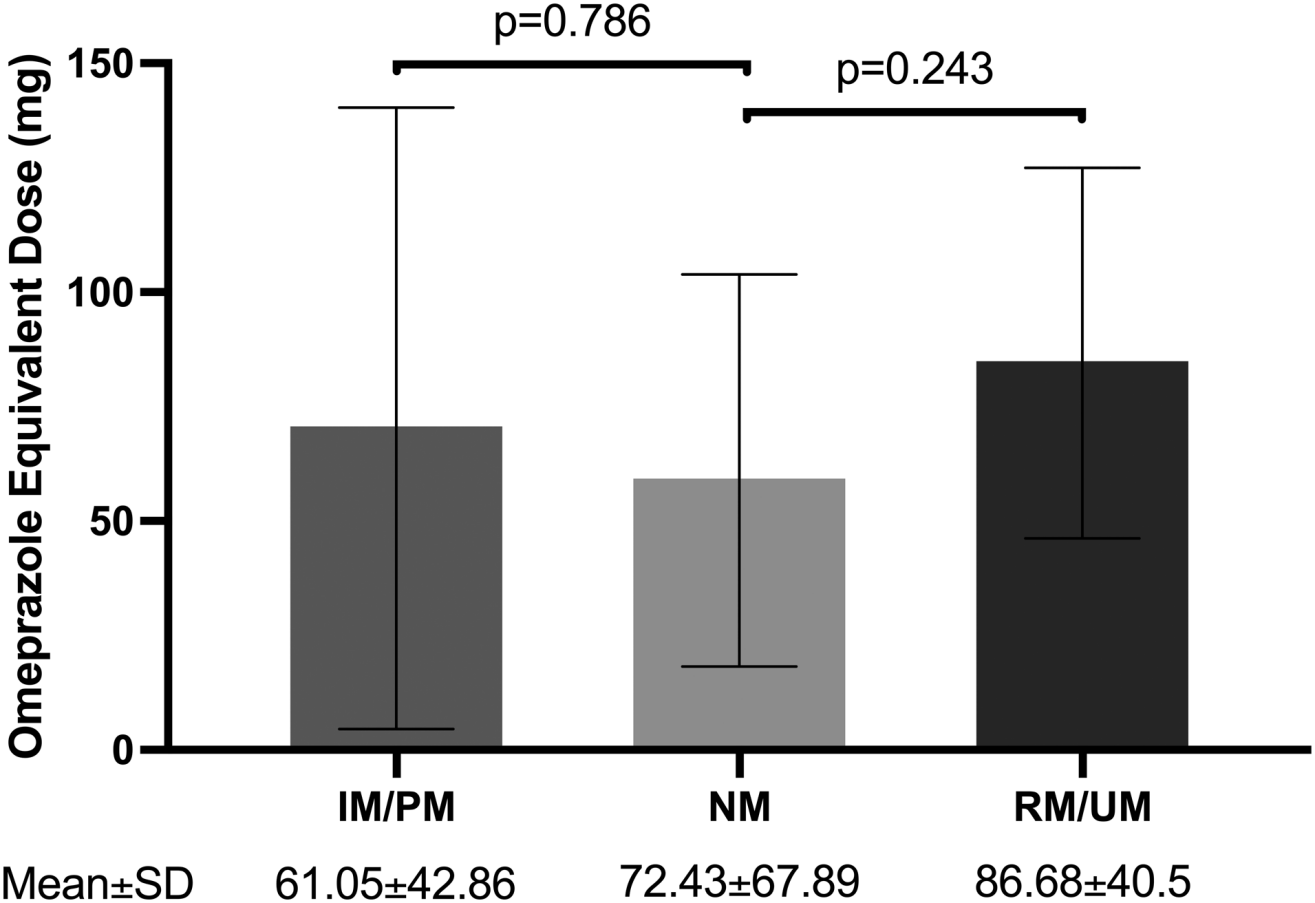
Proton-pump inhibitor (PPI) dosage across different CYP2C19 metabolizer groups. Information regarding PPI dosage was gathered on the day of *CYP2C19* genotyping, and all total daily dosages were later standardized to omeprazole equivalents (OEs) to facilitate uniformity and comparability. No significant differences in terms of OE PPI dosage were observed among the three groups. Error bars are included to show one standard deviation from the mean. SD, Standard Deviation.

No difference in terms of OE PPI dosage was observed on the day of *CYP2C19* genotyping when compared to the NM group (Figure 1). The mean OE dose for the NM group was 61.05 mg (SD = 42.86). In the IM/PM group, the mean OE dose was 72.43 mg (SD = 67.89). For the RM/UM group, the mean OE dose was 86.68 mg (SD = 40.5).

### Endoscopic and histologic findings

**Figure 2.**
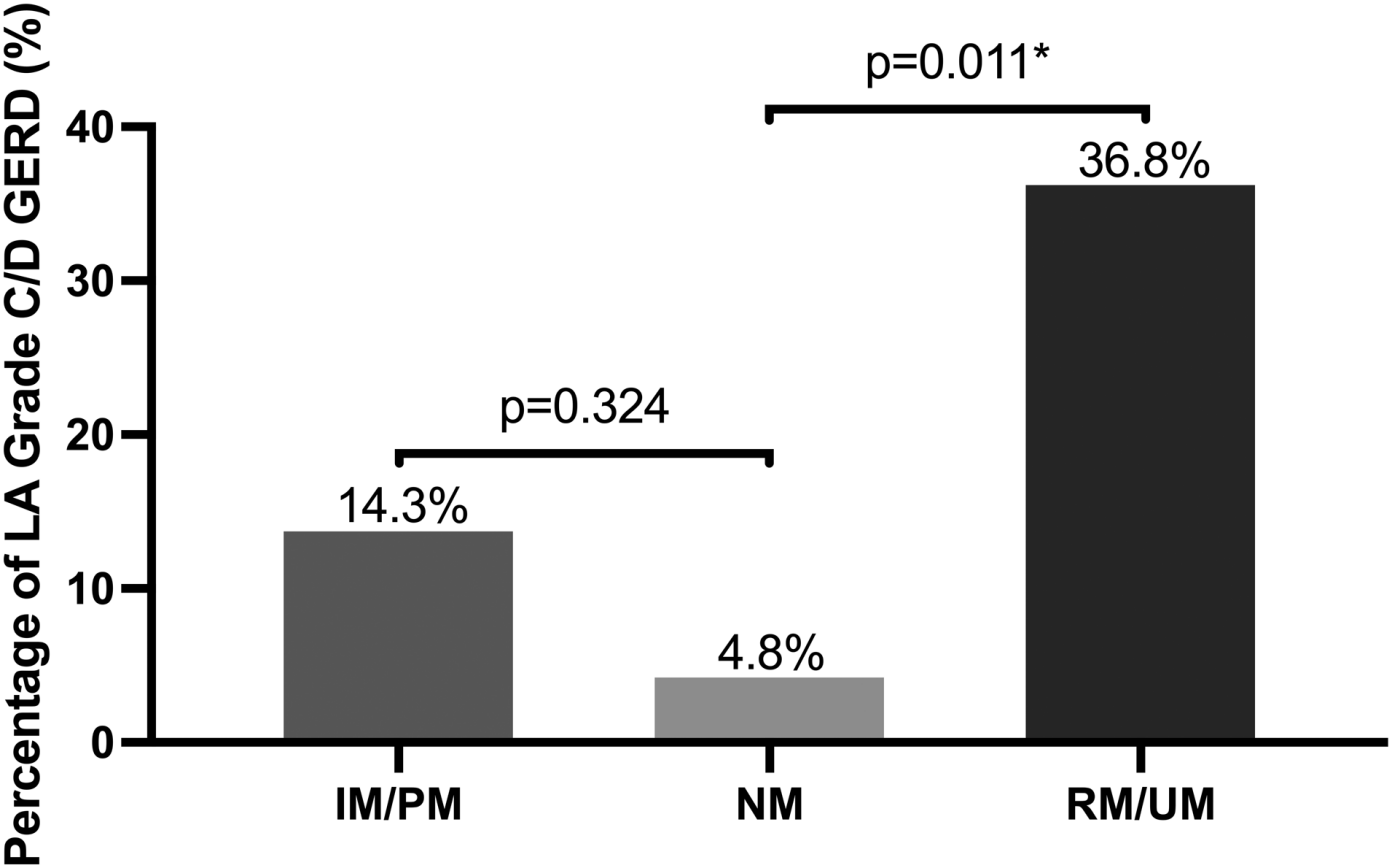
The proportion of patients displaying endoscopic evidence of LA grade C or D GERD within various CYP2C19 metabolizer groups, with bars representing the respective percentages of each group. Notably, the RM/UM group exhibited a significantly higher proportion of patients with endoscopic evidence of LA grade C or D GERD compared to the NM group. There was no difference between the IM/PM group and the NM group. An asterisk (*) denotes statistical significance, with a *P*-value of less than 0.05.

**Figure 3.**
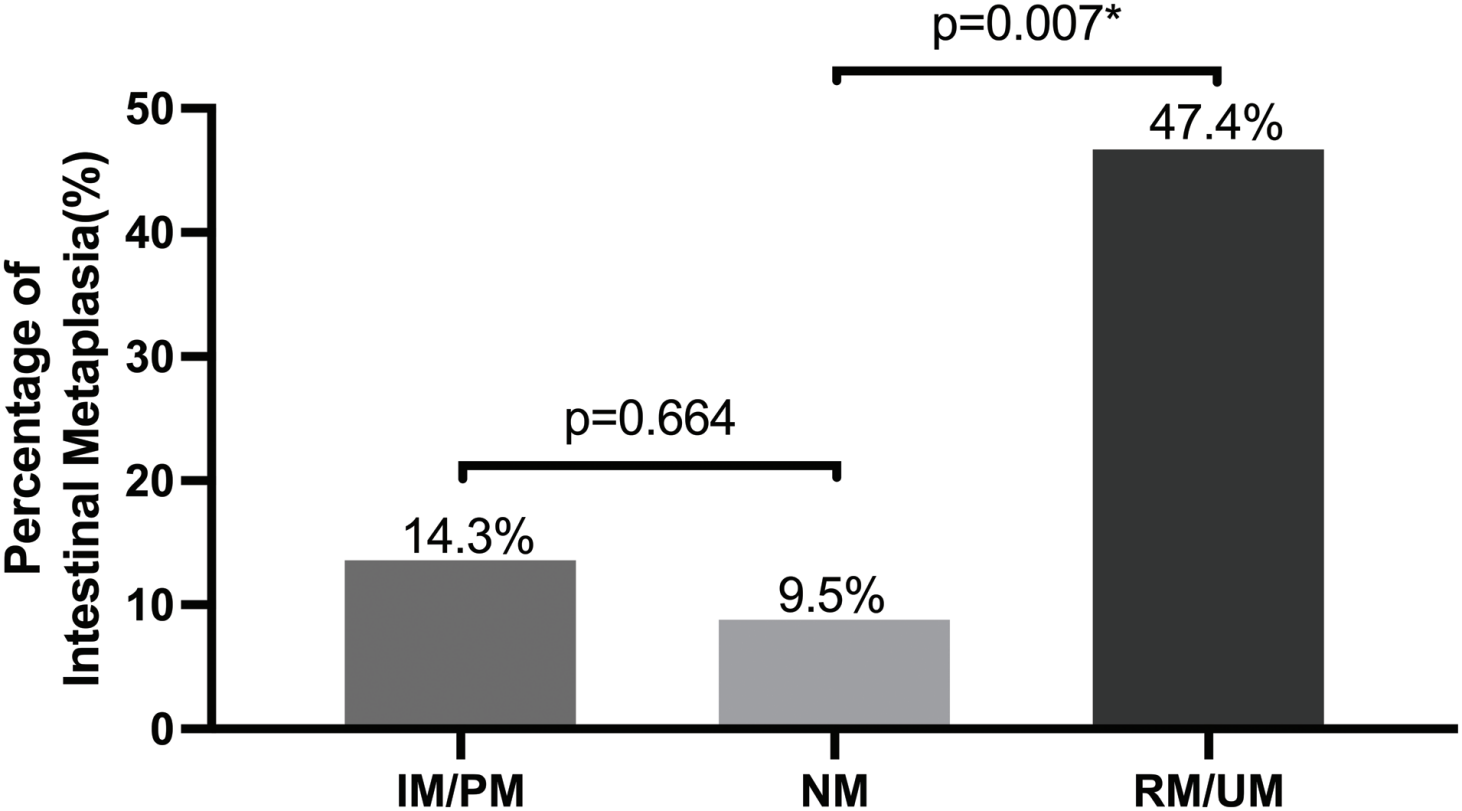
The proportion of patients displaying histologic evidence of intestinal metaplasia within various CYP2C19 metabolizer groups, with bars representing the respective percentages of intestinal metaplasia for each group. Notably, the RM/UM group exhibited a significantly higher proportion of patients with histologic evidence of intestinal metaplasia compared to the NM group. There was no difference between the IM/PM group and the NM group. An asterisk (*) denotes statistical significance, with a *P*-value of less than 0.05.

**Figure 4.**
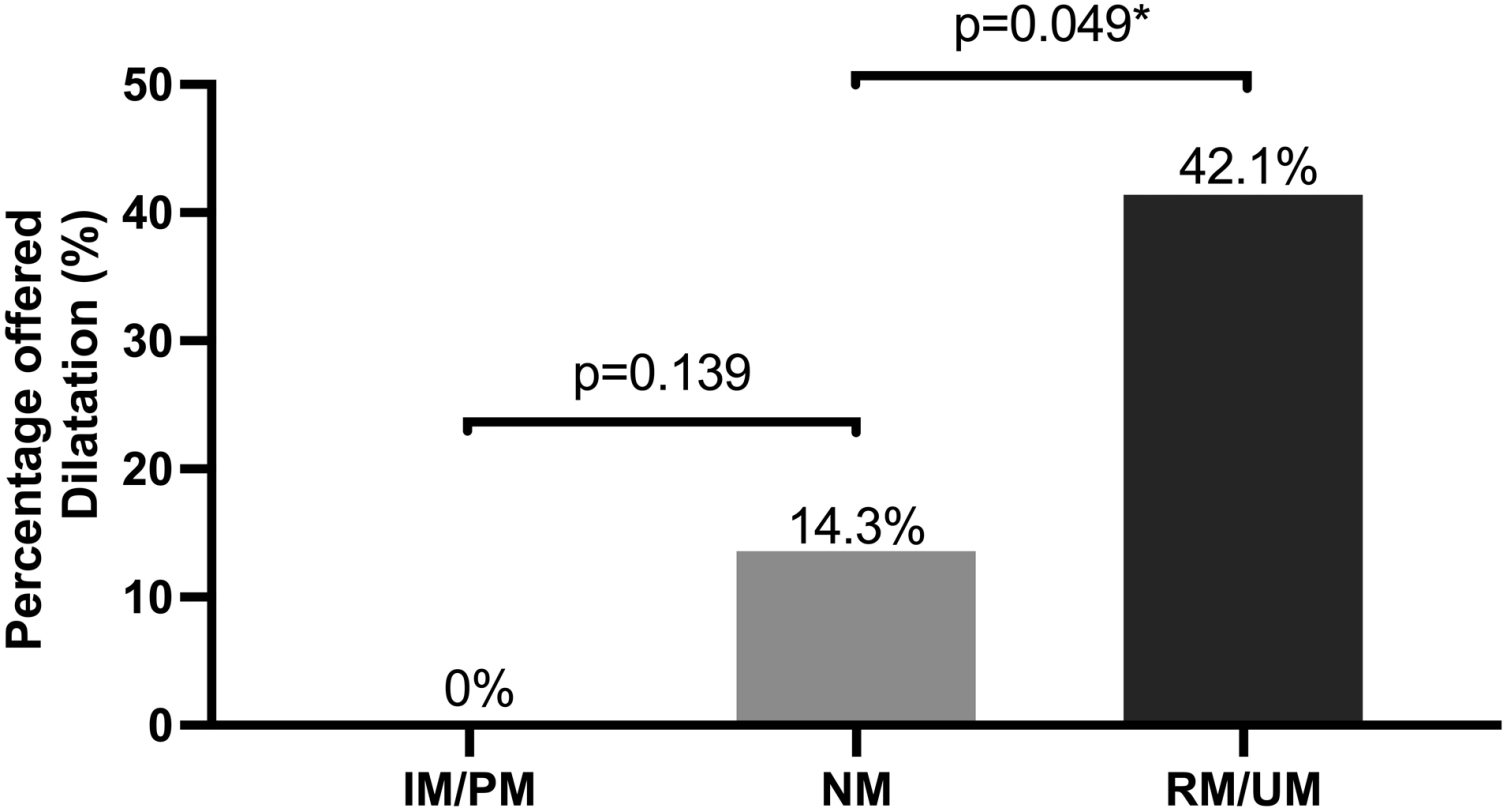
The proportion of patients being offered dilatation to relieve nonobstructive dysphagia within various CYP2C19 metabolizer groups, with bars representing the respective percentages of dilatation for each group. Notably, the RM/UM group exhibited a significantly higher proportion of patients being offered dilatation to relieve nonobstructive dysphagia compared to the NM group. There was no difference between the IM/PM group and the NM group. An asterisk (*) denotes statistical significance, with a *P*-value of less than 0.05.

The RM/UM group more frequently had endoscopic evidence of LA grade C or D GERD compared to the NM group (7/19, 36.8% vs 1/21, 4.8%, *P* = 0.011); no difference was observed between the IM/PM group and the NM group (Figure 2). The RM/UM group had a higher incidence of histologic evidence of intestinal metaplasia of the esophagus compared to the NM group (9/19, 47.4% vs 2/21, 9.5%, *P* = 0.007); no difference was observed between the IM/PM group and the NM group (Figure 3). A greater number of patients in the RM/UM group were offered dilatation of the esophagus for relief of nonobstructive dysphagia compared to the NM group (8/19, 42.1% vs 3/21, 14.3%, *P* = 0.049); no difference was observed between the IM/PM group and the NM group. (Figure 4).

### Ambulatory pH study

As shown in Table 2, the three groups exhibited similar DeMeester scores. It is important to note that all patients had stopped PPIs for 72 hours preceding the ambulatory pH study. The mean DeMeester score for the NM group was 21.46 (SD = 20.85, 95% confidence interval (CI): [11.97, 30.95]). The mean DeMeester score for the IM/PM group was 25.86 (SD = 31.91, 95% CI: [7.43, 44.28]). Lastly, the mean DeMeester score for the RM/UM group was 31.66 (SD = 38.99, 95% CI: [12.87, 50.46]). The *P*-value among the three groups was 0.587. The severity of GERD was categorized according to the value of DeMeester scores^16^; no differences were observed regarding the degree of severity of GERD among the three groups.

**Table 2.** DeMeester scores.

		NM group	IM/PM group	RM/UM group	*P*-value
DeMeester scores	Mean	21.46	25.86	31.66	0.587
	SD	20.85	31.91	38.99	
	95% CI for Mean	[11.97,30.95]	[7.43,44.28]	[12.87,50.46]	N/A
Severity of GERD	Normal	9 (42.86%)	8 (57.14%)	7 (36.84%)	0.696
	Mild	10 (47.62%)	5 (35.71%)	9 (47.37%)	
	Moderate	2 (9.52%)	0 (0%)	2 (10.52%)	
	Severe	0 (0%)	1 (7.14%)	1 (5.26%)	

The three groups exhibited similar DeMeester scores calculated from the ambulatory pH study. It is important to note that all patients had stopped PPI usage for 72 hours preceding the ambulatory pH study. The normal range for DeMeester score should be <14.72. A score falling within the range of 14.72 to 50 is indicative of mild GERD, while a score within the range of 51 to 100 is indicative of moderate GERD. A DeMeester score surpassing 100 is indicative of severe GERD. SD, standard deviation. CI, confidence interval. N/A, not applicable.

As further shown in Table 3, the RM/UM group exhibited a higher number of reflux episodes in the supine position. Specifically, the RM/UM group recorded a mean of 36.89 reflux episodes when supine, while the IM/PM group and NM group reported mean supine reflux episodes of 4.64 and 7.86, respectively. The *P*-value across the three groups was 0.006.

**Table 3.** All parameters from ambulatory pH study.

		NM group	IM/PM group	RM/UM group	*P*-value
Total esophageal pH < 4 Time (%)	Total	5.98 ± 9.69	6.94 ± 9.13	7.78 ± 10.17	0.843
	Upright	6.72 ± 9.69	5.39 ± 5.89	6.23 ± 6.91	0.888
	Supine	2.45 ± 3.76	5.72 ± 14.72	9.76 ± 17.62	0.219
	Post-prandial	7.09 ± 7.63	7.52 ± 7.98	7.88 ± 7.87	0.951
Number of reflux episodes	Total	64.38 ± 33.03	58.29 ± 41.06	92.84 ± 73.48	0.123
	Upright	57 ± 31.64	53.86 ± 8.49	56.58 ± 43.16	0.968
	Supine	7.86 ± 8.27	4.64 ± 7.06	36.89 ± 52.72	0.006*
	Post-prandial	34.05 ± 28.44	35.14 ± 28.31	40.11 ± 31.15	0.795
Number of reflux episodes ≥5 minutes	Total	2.1 ± 2.97	1.5 ± 2.07	4.05 ± 6.65	0.223
	Upright	1.67 ± 2.97	0.79 ± 1.48	1.42 ± 2.55	0.593
	Supine	0.43 ± 0.87	0.71 ± 1.64	2.63 ± 5.15	0.081
	Post-prandial	0.9 ± 0.89	0.57 ± 0.85	1.32 ± 1.97	0.302
Longest reflux (minutes)	Total	19.18 ± 38	47.18 ± 74.4	13.76 ± 12.67	0.096
	Upright	13.79 ± 36.65	25.34 ± 55.48	6.57 ± 7.98	0.351
	Supine	7.8 ± 15.49	23.32 ± 59.78	11.09 ± 13.38	0.376
	Post-prandial	7.04 ± 8.05	14.95 ± 31.53	6.07 ± 6.76	0.296

This table comprised all parameters obtained from ambulatory pH studies.

An asterisk (*) denotes statistical significance, with a P-value of less than 0.05.

### High-resolution esophageal manometry

**Figure 5.**
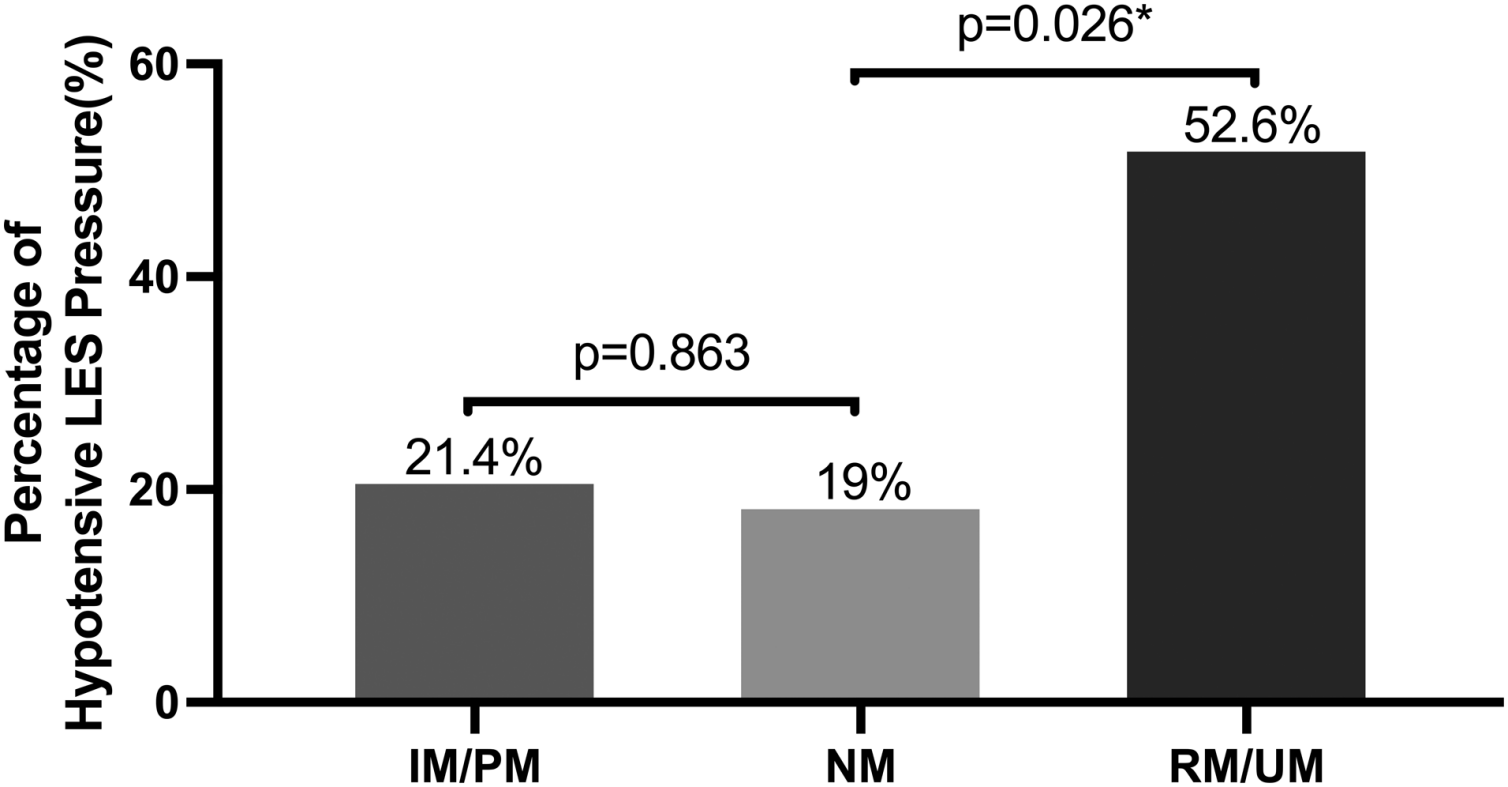
The proportion of patients displaying hypotensive lower esophageal sphincter (LES) resting pressure within various CYP2C19 metabolizer groups, with bars representing the respective percentages of hypotensive LES resting pressure for each group. Notably, the RM/UM group exhibited a significantly higher proportion of patients with hypotensive LES resting pressure compared to the NM group. There was no difference between the IM/PM group and the NM group. An asterisk (*) denotes statistical significance, with a *P*-value of less than 0.05.

On HRM, although having similar mean LES resting pressure, the RM/UM group more frequently exhibited a hypotensive LES resting pressure compared to the NM group (10/19, 52.6% vs 4/21, 19%, *P* = 0.026) (Figure 5). As shown in Table 4, the mean LES resting pressure for the NM group was 22.69 mmHg (SD = 14.96, 95% CI: [15.88, 29.51]). The mean LES resting pressure for the IM/PM group was 22 mmHg (SD = 13.39, 95% CI: [14.26, 29.74]). The mean LES resting pressure for the RM/UM group was 14.79 mmHg (SD = 13.79, 95% CI: [8.14, 21.45]). No significant difference was observed in the incidence of hiatal hernia among the three groups. In certain cases, data for DWA, onset velocity, DCI, and DL were unavailable. Specifically, one individual from the NM group (1/21, 4.76%), four from the PM/IM group (4/14, 28.57%), and four from the RM/UM group (4/19, 21.05%) had missing data for these parameters. As a result, these patients were excluded from the calculations involving DWA, onset velocity, DCI, and DL. While statistical differences were observed in DWA and DCI among the three groups, these measurements remained within the normal range. No significant differences were found in terms of onset velocity and DL.

**Table 4.** High-resolution esophageal manometry.

		NM group	IM/PM group	RM/UM group	*P*-value
Presence of H-hiatal hernia		7 (33.3%)	3 (21.4%)	7 (36.6%)	0.624
Lower esophageal sphincter resting pressure (mmHg)	Mean ± SD	22.69 ± 4.96	22 ± 13.39	14.79 ± 13.79	0.177
	95% CI for Mean	[15.88,29.51]	[14.26,29.74]	[8.14,21.45]	N/A
Distal wave amplitude (mmHg)	Mean ± SD	95.8 ± 38.13	77.67 ± 29.05	65.52 ± 14.55	0.017*
	95% CI for Mean	[77.97,113.67]	[58.16,97.19]	[57.46,73.58]	N/A
Onset velocity (cm/s)	Mean ± SD	3.73 ± 1.23	9.15 ± 16.15	3.6 ± 1.42	0.143
	95% CI for Mean	[3.15,4.3]	[-2.4,20.7]	[2.82,4.39]	N/A
Distal contraction integral (mmHg-cm-s)	Mean ± SD	1478.86 ± 826.81	677.21 ± 347.7	739.8 ± 360.54	<0.001*
	95% CI for Mean	[1091.9,1865.82]	[428.48,925.94]	[540.14,939.46]	N/A
Distal latency (seconds)	Mean ± SD	6.6 ± 1.36	9.26 ± 12.35	7.14 ± 1.61	0.501
	95% CI for Mean	[5.96,7.24]	[0.43,18.09]	[6.25,8.04]	N/A

The normal range for each result is as follows: lower esophageal sphincter resting pressure: 13-43 mmHg; distal wave amplitude: 43-152 mmHg; onset velocity: 2.8-6.3 cm/s; distal contraction integral: 450-8000 mmHg-cm-s; distal latency: >4.5 seconds.

An asterisk (*) denotes statistical significance, with a *P*-value of less than 0.05. SD, standard deviation; CI, confidence interval; N/A, not applicable.

## Discussion

To the best of our knowledge, our study represented the first to investigate both the extent of esophageal acid exposure from ambulatory pH studies and esophageal motor function from HRMs in individuals on chronic PPI therapy with refractory GERD symptoms belonging to different CYP2C19 metabolizer groups.

For individuals with GERD despite adhering to the standard once-daily PPI dosage, one of the alternative approaches is to double the PPI dose which showed overall improvement of symptoms.^17,18^ Additional PPI dose escalations have been documented in previous studies, with doses reaching as high as 64 mg of OE administered twice daily reported.^13^ In our study, mean OEs exceeded the standard omeprazole dosage in all three groups. We attributed this finding to the refractory nature of our patients, many of whom were on high PPI doses; specifically, 17 patients were prescribed dexlansoprazole 60mg daily (OE 110 mg), and 3 patients were on a daily 120 mg lansoprazole dose (OE 108 mg). The highest recorded dose was esomeprazole 80 mg twice daily by 1 patient (OE 256 mg).

Our study revealed that DeMeester scores did not significantly differ among different *CYP2C19* genotypes. It is important to note that this observation may have been influenced by the discontinuation of PPIs 72 hours prior to the pH study. However, we observed that the RM/UM group showed a higher number of reflux episodes when in supine. This finding suggested that the RM/UM group might be more symptomatic despite having comparable DeMeester scores. This difference, however, did not reflect on the DeMeester scores, as the number of reflux episodes when supine is not part of the six parameters that constitute the DeMeester score.^19^ In a small study involving 12 patients, it was observed that after a 14-day course of esomeprazole treatment, the duration of pH > 4 recorded in 24-hour monitoring did not differ significantly across different phenotypes.^4^ Conversely, another study demonstrated that CYP2C19 UMs displayed a statistically significant increase in duration during which the pH remained below 4.^20^ For future studies, it would be interesting to compare DeMeester scores among different metabolizer groups while on PPI therapy using a larger patient population.

Our study showed that despite having similar self-reported symptoms on the day of *CYP2C19* genotyping, RM/UM patients had more objective evidence of mucosal inflammation. Studies have shown that clinical symptoms of GERD have a poor correlation with actual endoscopic findings.^21–23^ Previous researches indicated that RM individuals experience a higher prevalence of residual symptoms following PPI treatment^24^ and more were also found to be associated with peptic ulcer diseases.^25–27^ Our study identified additional esophageal findings contributing to the refractiveness: despite receiving chronic PPI therapy, RMs and UMs exhibit poorer endoscopic and histologic outcomes. This further highlights that implementation of *CYP2C19* genotype-guided dosing is crucial in these patients, as PPIs are effective in promoting esophageal healing.^28^

In the absence of radiographic or endoscopic evidence of stenosis, endoscopists commonly employ empirical esophageal dilatation to alleviate symptomatic non-obstructive dysphagia.^29,30^ Although esophageal dilatation provides relief for non-obstructive dysphagia, its management remains controversial.^30^ In our study, the RM/UM group were more likely to undergo dilatation for symptom relief of nonobstructive dysphagia. At our center, empirical dilatation is often offered when symptoms are present without endoscopic evidence of stenosis or stricture. Unfortunately, no dysphagia scores were obtained before and after dilatation, making it challenging to quantify the severity of symptoms and assess the degree of improvement post-dilatation. Nevertheless, we wish to highlight that RM/UM patients may be generally more symptomatic despite being on chronic PPI therapy. In a previous case report, an RM individual was identified to have a rapid-evolving extensive esophageal stricture that necessitated additional surgical intervention.^31^ The above findings implied that RM/UM group may have been less responsive to conventional GERD treatment, necessitating more invasive measures to alleviate discomfort and improve esophageal function.

It has been reported that GERD can manifest in two distinct patterns: the first, with symptoms predominantly during the daytime or when in an upright position, is associated with transient lower esophageal sphincter relaxations (TLESR); and the second, with symptoms predominantly at night or when in a supine position, is associated with basal hypotensive LES pressure.^32^ In our study, we observed that the RM/UM group exhibited a higher number of reflux episodes when supine and more individuals were found to have hypotensive LES pressures. Previous studies have suggested that while esophageal motor impairment could be independent of the extent of esophagitis, the severity of mucosal inflammation might be inversely related to LES resting pressure and correlated with the frequency and duration of reflux.^33^ Specifically, as mucosal injury progresses, the prevalence of a mechanically defective LES increases.^33,34^ We believe that this is consistent with our findings, where the RM/UM group exhibited more mucosal inflammation, inversely affecting LES pressure. RM/UM patients had more reflux episodes in the supine position, possibly due to the higher frequency of hypotensive LES pressures, although this was not consistent across other parameters in the supine position.

### Limitations and strengths

This study has potential limitations outlined as follows:

(1) This retrospective single-center study, limited by a small sample size, may not fully represent the broader population. The limited sample size resulted from the constraint that only a small number of patients undergoing *CYP2C19* genotyping also participated in both ambulatory pH studies and HRMs, thereby restricting their eligibility for inclusion in the study.
(2) Despite previous studies that have demonstrated that RM/UM individuals typically require higher PPI doses to enhance the likelihood of efficacy, due to their lower plasma exposure levels,^6^ our study revealed comparable OE doses among all three groups. It is important to note that the data collected was obtained on the day of *CYP2C19* genotyping, suggesting that at the time of patient assessment for *CYP2C19* genotyping, the prescribed PPI doses did not correlate with predicted PPI metabolism.
(3) All patients had stopped PPI usage for 72 hours preceding the ambulatory pH, hindering our ability to ascertain any significant differences in the extent of acid exposure under conditions of acid suppression and to verify that symptoms of GERD on chronic PPI were actually due to acid reflux.
(4) Although we noticed that the RM/UM group had a higher number of reflux episodes when supine, this pattern was not consistent across other parameters (total esophageal pH < 4 time, number of reflux episodes ≥5 minutes, and longest reflux) in the supine position. It is therefore challenging to ascertain whether this has clinical significance or is merely an isolated numerical variation.
(5) Dysphagia scores were not collected before and after empirical esophageal dilatation for symptomatic non-obstructive dysphagia. There may be significant variation among different endoscopists when offering esophageal dilatation for non-obstructive dysphagia.^30^
(6) Data for DWA, onset velocity, DCI, and DL were unavailable for a subset of patients, potentially precluding the accuracy of the final results.
(7) A large proportion of our patients used second-generation PPIs. However, according to the CPIC guideline for *CYP2C19* and PPI dosing, the evidence linking *CYP2C19* genotype with variability in drug serum concentrations and effectiveness of second-generation PPIs is lacking due to the limited number of studies and the strength of the association.^6^

Despite these limitations, to the best of our knowledge, our study comprised the largest patient cohort encompassing various *CYP2C19* genotypes who underwent both ambulatory pH studies and HRMs. Our study also shed light on the complex interplay between CYP2C19 metabolizer status, chronic PPI therapy, and the severity of GERD.

## Conclusion

Our study revealed CYP2C19 RMs and UMs with refractory GERD symptoms despite undergoing chronic PPI therapy, exhibited more pronounced esophageal mucosal inflammation both endoscopically and histologically. Despite exhibiting similar acid exposure when PPIs were discontinued, RMs and UMs had a higher likelihood of being offered dilatation for symptom relief and more frequently found to have hypotensive basal LES pressures. This highlights the importance of seeking optimal PPI dosing in such patients to promote esophageal healing. Recognizing the need for tailored approaches, our study advocates for the consideration of *CYP2C19* genotype-guided PPI dosing as a potential standard of care for all GERD patients.

## Supplementary Material

gwae005_suppl_Supplementary_Materials

## Data Availability

The data underlying this article will be shared on reasonable request to the corresponding author.
